# Specific oral tolerance induction (SOTI) to cow’s milk in an adult patient with anaphylaxis symptoms

**DOI:** 10.1186/2045-7022-1-S1-P54

**Published:** 2011-08-12

**Authors:** José Geraldo Dias, Ana Célia Costa, Elisa Pedro, Manuel Pereira Barbosa

**Affiliations:** 1 Hospital Santa Maria, Centro Hospitalar Lisboa Norte, Immunoallergology, Lisbon, Portugal

## Introduction

Cow's milk allergy (CMA) in adults is less frequent and tend to persist longer than in children. SOTI is a valid treatment option for patients with persistent food allergy.

## Case report

A 20-year-old man, with no personal history of atopic disease, reported at 19 years his first episode of anaphylaxis (conjunctival hyperemia, generalized pruritus, oropharyngeal obstruction sensation, dyspnea and dysphagia) 30 minutes after ingestion of 150ml of milk and bread with butter. Since then, he mentioned oropharyngeal soreness whenever he ingested milk. One year later, he had a similar episode of anaphylaxis, 10 minutes after ingestion of a cheese/ham sandwish. He was recommended to avoid any foods that contain milk or its derivatives, but 2 months later, suffered the same reaction 1 hour after pizza ingestion. He self-administered ebastine (10mg) and prednisolone (40mg) with symptomatic relief within 1h. He denied any physical exercise or drug ingestion before the episodes, known drug or food sensitivity, and reported no symptoms with any kind of food in the past. SPT to aeroallergens: negative; to cow's milk and its proteins: positive. Specific IgE (KU/L) positive to: cow's milk-3.6; casein-7.7; alpha-lactoalbumin-1.8; beta-lactoglobulin-1.2. Total IgE-136U/mL. It was prescribed self-injectable epinephrine, avoidance of milk and its derivatives and the patient was proposed to cow’s milk SOTI. He reported on the first day’s protocol, oropharyngeal pruritus with spontaneous resolution, after each administration (cumulative dose-32ml). He achieved the end of the protocol (200ml daily) with no other symptoms. Six months later he had no dietary restrictions and did not report other episodes. SPT were performed after 6 months and revealed a reduction in milk skin test reactivity.

## Discussion

IgE-mediated CMA was confirmed. Avoiding milk and carrying self-injectable epinephrine are the current strategies for its management. SOTI to milk in this patient allowed a diet without restrictions (increased threshold dose for allergic reactions) and a substantial reduction in the risk of severe allergic reactions after inadvertent ingestion of milk or its derivatives.

